# Final analysis of randomized phase II study optimizing melphalan, prednisolone, bortezomib in multiple myeloma (JCOG1105)

**DOI:** 10.1111/cas.15484

**Published:** 2022-07-31

**Authors:** Dai Maruyama, Shinsuke Iida, Ryunosuke Machida, Shigeru Kusumoto, Noriko Fukuhara, Nobuhiko Yamauchi, Kana Miyazaki, Makoto Yoshimitsu, Junya Kuroda, Norifumi Tsukamoto, Hideki Tsujimura, Kensuke Usuki, Takahiro Yamauchi, Takahiko Utsumi, Ishikazu Mizuno, Yasushi Takamatsu, Yasuyuki Nagata, Shuichi Ota, Eiichi Ohtsuka, Ichiro Hanamura, Yasuhiro Suzuki, Shinichiro Yoshida, Satoshi Yamasaki, Youko Suehiro, Yutaro Kamiyama, Suguru Fukuhara, Kunihiro Tsukasaki, Hirokazu Nagai

**Affiliations:** ^1^ Department of Hematology Oncology, Cancer Institute Hospital Japanese Foundation for Cancer Research Tokyo Japan; ^2^ Department of Hematology and Oncology Nagoya City University Hospital Nagoya Japan; ^3^ JCOG Data Center National Cancer Center Hospital Tokyo Japan; ^4^ Department of Hematology and Rheumatology Tohoku University Hospital Sendai Japan; ^5^ Department of Hematology National Cancer Center Hospital East Kashiwa Japan; ^6^ Department of Hematology and Oncology Mie University School of Medicine Tsu Japan; ^7^ Department of Hematology and Rheumatology Kagoshima University Hospital Kagoshima Japan; ^8^ Division of Hematology and Oncology Kyoto Prefectural University of Medicine Kyoto Japan; ^9^ Department of Hematology Gunma University Hospital Maebashi Japan; ^10^ Division of Hematology‐Oncology Chiba Cancer Center Chiba Japan; ^11^ Department of Hematology NTT Medical Center Tokyo Tokyo Japan; ^12^ Department of Hematology and Oncology University of Fukui Fukui Japan; ^13^ Department of Hematology Shiga General Hospital Moriyama Japan; ^14^ Department of Hematology Hyogo Cancer Center Akashi Japan; ^15^ Division of Medical Oncology, Hematology and Infectious Diseases Fukuoka University Hospital Fukuoka Japan; ^16^ Department of Internal Medicine III Hamamatsu University School of Medicine Hamamatsu Japan; ^17^ Department of Hematology Sapporo Hokuyu Hospital Sapporo Japan; ^18^ Department of Hematology Oita Prefectural Hospital Oita Japan; ^19^ Division of Hematology Aichi Medical University Nagakute Japan; ^20^ Department of Hematology National Hospital Organization Nagoya Medical Center Nagoya Japan; ^21^ Department of Hematology National Hospital Organization Nagasaki Medical Center Ohmura Japan; ^22^ Department of Hematology and Clinical Research Institute National Hospital Organization Kyushu Medical Center Fukuoka Japan; ^23^ Department of Hematology National Hospital Organization Kyushu Cancer Center Fukuoka Japan; ^24^ Department of Clinical Oncology and Hematology The Jikei University Hospital Tokyo Japan; ^25^ Department of Hematology National Cancer Center Hospital Tokyo Japan; ^26^ Department of Hematology, International Medical Centre Saitama Medical University Saitama Japan

**Abbreviations**
AE: adverse event
CI: confidence interval
CR: complete response
HR: hazard ratio
JCOG: Japan Clinical Oncology Group
MPB: melphalan, prednisolone, bortezomib
MRD: minimal residual disease
OS: overall survival
PFS: progression‐free survival
PS: performance status
TI‐NDMM: transplant‐ineligible newly diagnosed multiple myeloma

We conducted a randomized phase II study to determine a more promising modified MPB regimen for TI‐NDMM (JCOG1105, jRCTs031180097). The primary analysis in JCOG1105 revealed that Arm A (known as PETHEMA/GEM05 MPB) showed a higher CR rate and longer PFS without intolerable toxicities compared with Arm B (a further less intensive MPB) at a median follow‐up period of 26 months, suggesting that the twice‐weekly dosing of bortezomib in the first cycle along with a higher dose of melphalan and higher cumulative dose of both bortezomib and melphalan influenced the efficacy of the modified MPB regimen in patients with TI‐NDMM^1^ (Appendix S1). Here, we report the updated results from preplanned analysis of JCOG1105 with a 3‐year follow‐up from the end of accrual.

Between July 2013 and April 2016, in total 91 patients were randomized to Arm A (45 patients) and Arm B (46 patients). As for the data cut‐off (June, 2019), the median follow‐up period of all eligible patients was 47.3 months (range 10.4–71.1). The PFS rates at 1, 3, and 5‐years (95% CI) were 86.0% (71.6%–93.5%), 27.9% (15.6%–41.6%), and 16.4% (5.8%–31.8%) in Arm A, and 73.3% (57.8%–83.9%), 13.3% (5.4%–24.9%) and not estimable in Arm B with the HR of Arm B to Arm A being 1.69 (95% CI 1.06–2.68; Figure 1A). Predefined subgroup analyses of PFS are shown in Figure 2. Female patients seemed to have better PFS in Arm A (HR in Arms B to A, 2.87 [95% CI 1.34–6.61]) compared with male patients (HR in Arms B to A, 1.18 [95% CI 0.66–2.13]; Figure 2A,B), and patients with PS 2‐3 also showed a tendency to have better PFS in Arm A (HR in Arms B to A, 4.32 [95% CI 1.42–13.1]), unlike patients with PS 0–1 (HR in Arms B to A, 1.44 [95% CI 0.85–2.45]; Figure 2C,D). The OS rate at 5 years was 73.4% (95% CI 54.8%–85.3%) in Arm A and 56.8% (95% CI 31.2%–76.0%) in Arm B, respectively (HR in Arms B to A, 1.58 [95% CI 0.71%–3.53]) (Figure 1B). The OS was similar between Arms A and B. In total, 25 patients (10 in Arm A and 15 in Arm B) died during the follow‐up period, with a tendency toward numerical imbalance regarding death from myeloma (six in Arm A and 11 in Arm B). In comparison with AEs reported in the primary analysis,^1^ there were no marked changes in the incidence and severity of AEs reported in the final analysis.

**FIGURE 1:**
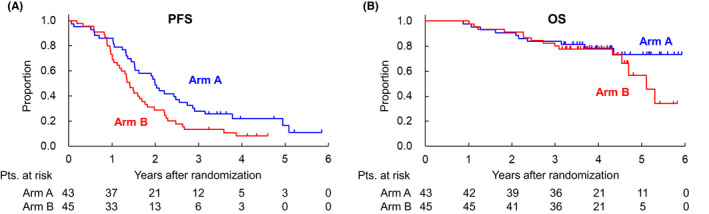
PFS and OS. (A) The 5‐year PFS was 16.4% (5.8%–31.8%) in Arm A, and not estimable in Arm B. (B) The 5‐year OS was 73.4% (95% CI 54.8%–85.3%) in Arm A and 56.8% (95% CI 31.2%–76.0%) in Arm B

**FIGURE 2:**
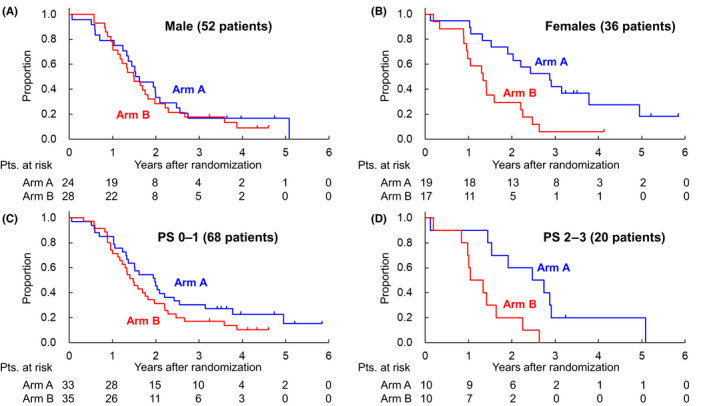
PFS of predefined subgroups. (A, B) Female patients seemed to have better PFS in Arm A unlike male patients. (C, D) PS 2–3 seemed to have better PFS in Arm A unlike PS 0–1

There are no reports regarding the influence of gender on the survival of patients with TI‐NDMM who were treated with a bortezomib‐containing regimen, and the reason why female patients seemed to have a better PFS in Arm A in the present study seemed to be unclear. Although there was a slight imbalance in the number of patients with International Staging System (ISS) stage III (14 males and two females) and expression of adverse chromosomal translocation‐associated genes (FGFR3 or MAF mRNA; three males and seven females), other patient characteristics, treatment exposure including percentage planned dose of bortezomib, melphalan, and prednisolone and incidence of AEs were similar between female and male patients. Among patients with PS 2–3 at study enrollment (10 patients each in both arms), the long‐term PFS in Arm A also tended to be better compared with that in Arm B. As our eligibility criteria permitted the enrollment of patients with PS 3 only resulting from osteolytic lesions (six patients in Arm A and eight patients in Arm B), rapid responses to treatment and improvement of patients' condition could have resulted in better PFS in Arm A.

In JCOG1105, although a higher median cumulative dose of melphalan was administered in Arm A (324 mg/m^2^) compared with in Arm B (252 mg/m^2^), a lower incidence of second primary malignancies was observed in Arm A (one patient) compared with in Arm B (five patients; Table 1). This result was consistent with the long‐term follow‐up findings of the VISTA study^2^ that showed no increased risk of second primary malignancies with MPB.

**TABLE 1:** Second primary malignancies

Arm	Second primary malignancy	Onset of second primary malignancy after protocol treatment	Subsequent treatment
Arm A	Early gastric cancer	214 days	None
Arm B	Esophageal cancer	31 months	Lenalidomide/dexamethasone
	Acute myeloid leukemia	26 months	Lenalidomide/dexamethasone
	Cutaneous squamous cell carcinoma	106 days	None
	Prostate cancer	23 months	Lenalidomide/dexamethasone
	Intramucosal gastric cancer	35 months	Lenalidomide/dexamethasone

In summary, the final analysis of JCOG1105 demonstrated that twice‐weekly dosing of bortezomib in the first cycle along with higher dose of melphalan and higher cumulative dose of both bortezomib and melphalan (Arm A) confers sustained PFS benefit with no new AE‐related concerns. However, a continued risk of relapse was observed in both arms because maintenance therapy was not recommended and all patients except two did not receive maintenance therapy in JCOG1105. Based on the results of this study, we are now conducting a next clinical trial incorporating anti‐CD38 antibody and fixed‐duration maintenance therapy combined with a modified MPB regimen and assessment of high‐risk cytogenetics and MRD (JCOG1911; jRCTs031200320).

## AUTHOR CONTRIBUTION

DM, SI, and RM conceived and designed the study; all authors provided study materials and recruited patients; DM, SI, and RM collected, analyzed, and interpreted the data; DM, SI, and RM wrote the manuscript; all authors gave final approval of the manuscript.

## FUNDING INFORMATION

This work was supported in part by the National Cancer Center Research and Development Funds (26‐A‐4, 29‐A‐3, and 2020‐J‐3), a Grant‐in‐Aid for Clinical Cancer Research (H26‐kakushin‐teki‐gan‐ippan‐074) from the Ministry of Health, Labour and Welfare of Japan, and by the Japan Agency for Medical Research and Development under grant nos. JP16ck0106077, JP19ck0106348 and 21ck0106685h0001 (DM).

## DISCLOSURE

DM reports honoraria (Janssen, Mundhipharma, Eisai, Chugai); research funding (Celgene, Novartis, Chugai, Ono, Takeda, Janssen, MSD). SI, an editorial board member of Cancer Science, reports honoraria (Celgene, Sanofi, Janssen, Takeda, Ono, Bristol Myers Squibb); research funding (Bristol Myers Squibb, Celgene, Janssen, Daiichi Sankyo, Amgen, Ono, AbbVie, GlaxoSmithKline, Eli Lilly, Caelum, Pfizer, Takeda, Sanofi). RM has nothing to disclose. SK reports honoraria (Chugai, Kyowa Kirin, Janssen, Daiichi Sankyo); research funding (Chugai, Kyowa Kirin, Janssen, Daiichi Sankyo). NF reports honoraria (Chugai, Kyowa Kirin, Huya Japan); research funding (AbbVie, Bayer, Chugai, Celgene, Eisai, Gilead, Incyte, Ono, Solasia). NY, KM, and MY have nothing to disclose. JK reports honoraria (Janssen). NT and HT have nothing to disclose. KU reports honoraria (Novartis Pharma); research funding (Astellas Pharma, AbbVie, Apellis, Symbio, Daiichi Sankyo, Novartis, Janssen, Otsuka, Astellas‐Amgen‐Biopharma, Takeda, Nippon Shinyaku, Bristol Myers Squibb). TY reports honoraria (Janssen). TU and IM have nothing to disclose. YT reports honoraria (Takeda, Janssen, Celgene); scholarship (Chugai, Takeda, Taiho, Astellas, Ono). YN has nothing to disclose. SO reports honoraria (Novartis, Bristol Myers Squibb); research funding (Kyowa Kirin). EO has nothing to disclose. IH reports honoraria (Bristol Myers Squibb, Takeda, Celgene, Sanofi, Janssen); research funding (Bristol Myers Squibb, Celgene). YS, SY, SY, YS, YK, and SF have nothing to disclose. KT reports honoraria (Daiichi Sankyo, Chugai, Byer, Kyowa Kirin, HUYA BIO, Bristol Myers Squibb). HN reports honoraria (Celgene, Esai, Chugai, Ono, Mundipharma); research funding (Bayer, AstraZeneca, Zenyaku Kogyo, Takeda, Mundipharma, SymBio, Chugai); scholarship (Chugai).

## ETHICS STATEMENT

*Approval of the research protocol*: The study protocol was approved by the Protocol Review Committee of JCOG, and was reviewed and approved by the National Cancer Center Hospital Certified Review Board (CRB3180008).

*Informed consent*: Written informed consent was obtained from all the patients.

*Registry and registration No. of the trial*: jRCTs031180097.

*Animal studies*: N/A.

## Supporting information


Appendix S1
