# Supplementary material for: Final analysis of randomized phase II study optimizing melphalan, prednisolone, bortezomib in multiple myeloma (JCOG1105)
Source: Cancer Sci. 2022 Jul 31;113(9):3267–70. doi: 10.1111/cas.15484 (PMC9459263; doi:10.1111/cas.15484)
Supplement: Supplementary file 1 — Appendix S1 [file CAS-113-3267-s001.docx]

**Supporting information**

**Study design and treatment regimens of JCOG1105**^1^


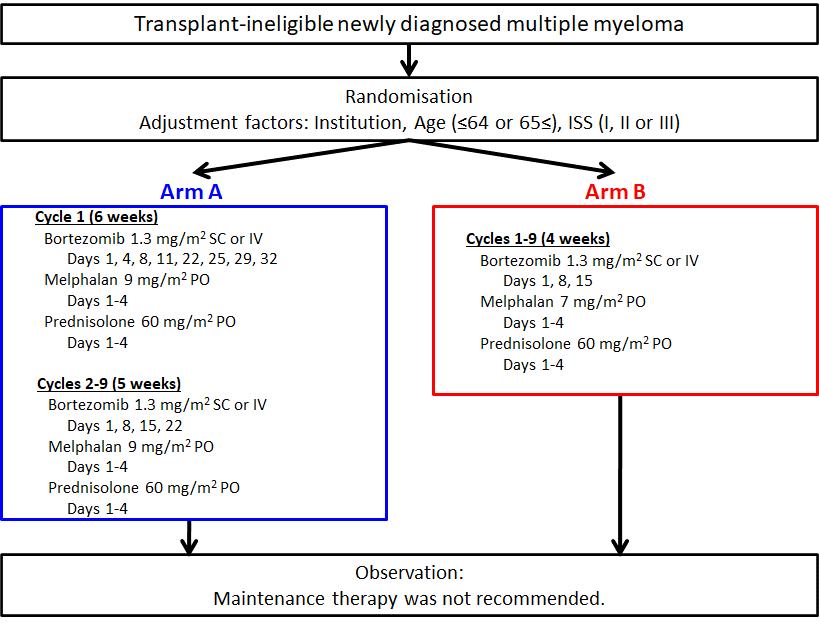


- Arm A consisted of one cycle of subcutaneous (SC) or intravenous (IV) bortezomib at 1.3 mg/m² administered twice-weekly plus 9 mg/m² of oral melphalan and 60 mg/m² of prednisolone on days 1-4 of a 6-week cycle, followed by eight 5-week cycles of 4-times once-weekly bortezomib plus the same doses of MP.
- Arm B consisted of nine 4-week cycles of SC or IV bortezomib at 1.3 mg/m² administered 3-times once-weekly plus 7 mg/m² of melphalan and 60 mg/m² of oral prednisolone on days 1-4.

1. Maruyama D, Iida S, Ogawa G, et al. Randomised phase II study to optimise melphalan, prednisolone, and bortezomib in untreated multiple myeloma (JCOG1105). Br J Haematol 2021;192(3):531–541.
